# A comparative study of bone density in elderly people measured with AI and QCT

**DOI:** 10.3389/frai.2025.1582960

**Published:** 2025-07-23

**Authors:** Min Guo, Yu Zhang, XinXin Gu, Xuhui Liu, Fei Peng, Zongjun Zhang, Mei Jing, Yingxia Fu

**Affiliations:** ^1^Affiliated Hospital of Integrated Traditional Chinese and Western Medicine, Nanjing University of Chinese Medicine, Nanjing, China; ^2^Jiangsu Province Academy of Traditional Chinese Medicine, Nanjing, China

**Keywords:** osteoporosis, bone density, quantitative QCT, artificial intelligence, BMD values

## Abstract

**Background:**

Osteoporosis, a systemic skeletal disorder characterized by deteriorated bone microarchitecture and low bone mass, poses substantial fracture risks to aging populations globally. Early detection of reduced bone mineral density (BMD) through opportunistic screening is critical for preventing fragility fractures. Although dual-energy X-ray absorptiometry (DXA) is the gold standard for diagnosing osteoporosis, many patients have not undergone screening with this technique. Therefore, developing an automated tool that can diagnose bone density through routine chest and abdominal CT examinations is highly important. With advancements in technology and the accumulation of clinical data, the role of bone density artificial intelligence (AI) in the diagnosis and management of osteoporosis is becoming increasingly significant.

**Objective:**

First to validate the diagnostic equivalence of AI-based BMD prediction against quantitative CT (QCT) reference standards, second to assess inter-device measurement consistency across multi-vendor CT systems (Siemens, GE, Philips). Ultimately, the objective is to determine the clinical utility of AI-derived BMD for osteoporosis classification.

**Methods:**

In this retrospective multicenter study, paired CT/QCT datasets from 702 patients (2019–2022) were analyzed. The accuracy, sensitivity, and specificity of an Bone Density AI model were evaluated by comparing the predicted bone mineral density values from bone density AI with the measured values from QCT. Moreover, the consistency of lumbar spine BMD measurements between QCT and Bone Density AI on different devices was compared.

**Results:**

The AUC of Bone Density AI model in diagnosing osteoporosis was 0.822 (95% CI: 0.787–0.867, *p* < 0.001), with an accuracy of 0.9456, sensitivity of 0.9601, and specificity of 0.9270, indicating good performance in predicting bone density. The consistency study between Bone Density AI and QCT for the vertebral BMD measurements revealed no statistically significant difference in *R*^2^ values, suggesting no significant difference in performance between the two methods in measuring BMD. The linear regression fit between the *R*^2^ values of QCT and Bone Density AI for measuring lumbar spine BMD with different equipment ranged from 0.88 to 0.96, indicating a high degree of consistency between the two measurement methods across devices.

**Conclusion:**

This multicenter study pioneers a dual-validation framework to establish the clinical validity of deep learning-based BMD prediction algorithms using routine thoracic/abdominal CT scans. Our data suggest that AI-driven BMD quantification demonstrates non-inferior diagnostic accuracy to QCT while overcoming DXA’s accessibility limitations. This technology enables cost-effective, radiation-free osteoporosis screening through routine CT repurposing, particularly beneficial for resource-constrained settings.

## 1 Introduction

Osteoporosis is the most prevalent bone disorder affecting aging populations, characterized by a disruption of the delicate balance between bone resorption and bone formation (Shevroja et al., 2023; Imamudeen et al., 2022). The subsequent loss of bone substance and changes in bone microarchitecture create an environment highly conducive to fractures, which are associated with high morbidity and mortality (McCloskey et al., 2021; Kaesmacher et al., 2017; Chen et al., 2022). Timely detection of presymptomatic bone loss remains paramount for implementing fracture prevention strategies and preserving musculoskeletal health.

Despite the high prevalence of osteoporosis and osteoporotic fractures, the current diagnosis rates, treatment rates, and preventive treatments for fragility fractures are inadequate (Cheng et al., 2021). One significant reason is the low utilization of bone density testing equipment and the relatively limited number of detection methods employed with these devices. In clinical practice, bone density is typically detected with dual-energy X-ray absorptiometry (DXA) and quantitative CT (QCT) devices. Many countries fail to meet the minimum service requirement of 11 units per million population for DXA proposed by the International Osteoporosis Foundation; in China, this number is extremely low, with less than 1 unit per million population (Clynes et al., 2020; Hsieh et al., 2021). Additionally, worldwide, the rates of DXA and Fracture Risk Assessment Tool (FRAX) use are low. In the United States, only 30% of women and 4% of men aged ≥65 years have their bone density measured with DXA. In Europe, only 24.9% of 70-year-old women have undergone bone density testing (Clynes et al., 2020; Kanis et al., 2021; Morden et al., 2014).

QCT opportunistic screening utilizing existing CT imaging protocols presents a cost-effective strategy for augmenting osteoporosis detection rates in unscreened populations. This approach eliminates the need for additional costs, time, or radiation exposure affected by factors such as obesity, osteophyte formation, or aortic calcification, thereby yielding a substantially improved sensitivity and accuracy in detecting osteoporosis (Sollmann et al., 2022). While QCT provides volumetric BMD quantification with superior diagnostic accuracy, its clinical implementation faces dual barriers: First technological limitations: Single-energy QCT requires specialized phantoms and proprietary software. Additional workflow integration challenges: <5% of routine abdominal CT scans currently incorporate opportunistic BMD analysis.

Recently, bone density AI technology has shown great potential in the early diagnosis, prevention, standardized treatment, and follow-up of osteoporosis. By combining low-dose CT scans with bone density AI measurement systems, bone density can be effectively measured, and the results are highly correlated and consistent with the results of the QCT measurements. Such AI-based bone density measurement systems not only successfully predict bone density in various populations but also have high accuracy, sensitivity, and specificity in diagnosing osteoporosis. However, there is currently a lack of comparative analysis data for these two methods, and the diagnostic performance of bone density AI technology requires strong data support.

This study is the first to comparative analysis of AI-derived vBMD against QCT reference standards (Mindways QCT Pro® calibration) across three major CT platforms (Siemens SOMATOM Force, GE Revolution CT, Philips IQon Spectral CT). This provides critical evidence for FDA/CE certification of device-agnostic Embeds osteoporosis detection into routine CT workflows without additional scanning. At the same timemaximizes screening capacity in DXA-underserved regions (<1 scanner/million population).

The importance of this study lies in its potential to provide a new, noninvasive, and efficient tool for the early diagnosis and treatment of osteoporosis, especially among populations that have not undergone osteoporosis screening. With deep learning technology, existing CT scan data can be fully utilized without the need for additional examinations or radiation exposure, thereby improving the diagnosis rate and timeliness of treatment for osteoporosis.

## 2 Materials and methods

### 2.1 Clinical data

A total of 625 elderly patients who underwent QCT bone density measurements at Nanjing Mingji Hospital from 2015 to 2019 and routine clinical abdominal CT scans at Jiangsu Province Hospital of Integrated Traditional Chinese and Western Medicine from 2020 to 2022 were included, including 192 who underwent physical examination at Mingji Hospital. The age of the patients ranged from 60 to 99 years (mean ± SD: 70.94 ± 8.47 years). Among the 625 patients, 320 were female (age 60–99 years, mean ± SD: 70.52 ± 7.98 years), and 305 were male (age 60–97 years, mean ± SD: 71.38 ± 8.94 years). All patients were free from systemic diseases affecting bone metabolism, such as chronic kidney disease and primary and secondary hyperparathyroidism, and had no history of taking medications affecting bone metabolism, such as corticosteroids.

### 2.2 Equipment and methods

CT examinations were performed with 16-slice (United Imaging uCT 510) and 64-slice (NeuViz 128, Neusoft Medical; GE Lightspeed VCT) CT scanners. The CT scan parameters included a fixed tube voltage of 120 kVp, automatic tube current, scan collimation of 16 × 0.6 mm and 64 × 0.625 mm, pitch of 0.9–1, rotation time of 0.5 s, table height of 135 cm, and field of view of 50 cm. Images were reconstructed with a standard soft tissue algorithm at a thickness of 1–1.5 mm. For the QCT bone density examination at Mingji Hospital, a solid phantom from Mindways, USA, was placed under the patient’s waist during the scan, covering vertebrae L2–L4. However, routine clinical abdominal examinations do not have synchronized calibration phantoms, and the scanning range includes the thoracic 12 lumbar 3 vertebrae.

QCT measurements were obtained with third-generation (Model 3 QCT pro v5.0, Mingji Hospital) and fourth-generation (Model 4 QCT pro v6.1) QCT software systems from Mindways. All images with a reconstruction thickness of 1–1.5 mm were uploaded to the QCT workstation, where two physicians measured the trabecular bone density of vertebrae T12 to L3 or L2 to L4 within the same period. The region of interest (ROI) was automatically outlined by the software and manually adjusted by the physicians to avoid cortical bone, bone islands, and the posterior venous groove while maintaining an ROI depth of 7–9 mm.

**Figure 1:**
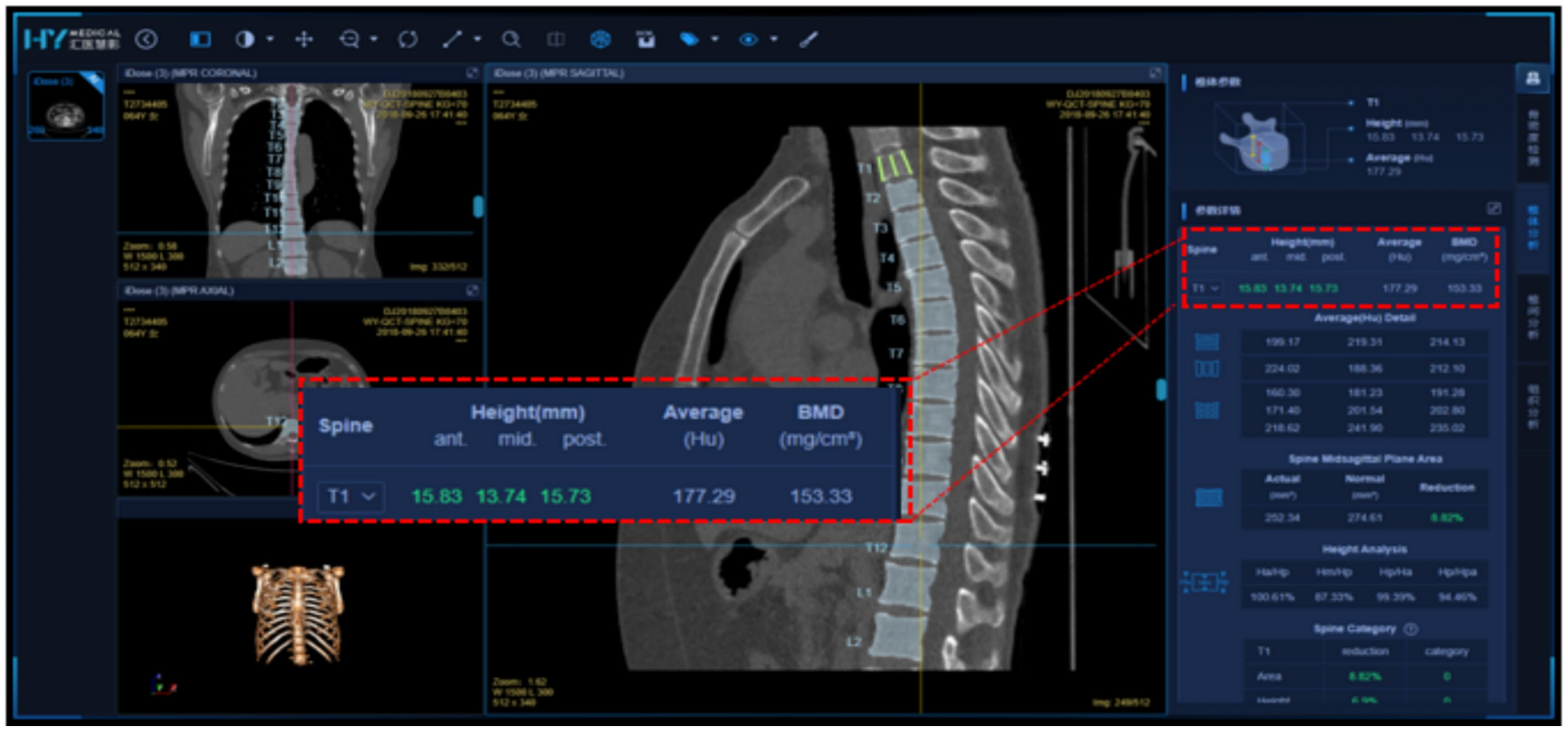
Detailed visualization interface of Huiyi Huiying AI quantitative CT bone density detection software.

Huiyi Huiying AI-based quantitative CT bone density measurement software, a commercially available deep learning-based quantitative CT auxiliary diagnostic program developed by Huiyi Huiying Company, was used for fully automated vertebral segmentation and extraction of vertebral BMD values. The software automatically recognizes and segments the thoracolumbar vertebrae; an example of the visual interface for the software is shown in Figure 1. As shown in Figure the software includes two main functions: vertebral segmentation and extraction of vertebral BMD values. CT images containing the lumbar spine are input into the software. The deep learning-based segmentation algorithm accurately locates and segments the T12 to L4 vertebrae. The segmentation results are then qualitatively judged by two physicians. After verification, the software directly extracts the average central CT values and trabecular bone density values of the T12, L1, L2, L3, and L4 vertebrae for each patient and records them. This section provides a detailed account of the study methodology, ensuring transparency and reproducibility of the research process (Figure 1).

### 2.3 Definitions

Osteoporosis: Diagnosed when the average BMD of the L1–L2 or L2–L3 vertebrae is less than 80 mg/cm^3^. Reduced bone mass: Defined as an average BMD of the L1–L2 or L2–L3 vertebrae between 80 and 120 mg/cm^3^. Normal bone density: Defined as an average BMD greater than 120 mg/cm^3^ for the L1–L2 or L2–L3 vertebrae.

Using the QCT measurements as the gold standard, the Bone Density AI quantitative judgment results are analyzed. Additionally, a comparative analysis of the QCT and bone density AI results for each vertebra from T12 to L4 was conducted. This approach allows a standardized assessment of the risk of osteoporosis on the basis of established medical guidelines, providing a clear framework for diagnosing and categorizing bone health with both QCT and AI technologies. The comparative analysis between QCT and AI measurements offers valuable insights into the accuracy and reliability of Bone Density AI in the context of the diagnosis of osteoporosis.

### 2.4 Statistical analysis

MedCalc 16.5 software was used for all the statistical analyses. The Kolmogorov–Smirnov test revealed that the age variable was normally distributed; this, variable is presented as the mean ± standard deviation. Categorical data (including sex) were compared with the chi-square test and are presented as frequencies (percentages). The average BMD of the L1–L2 or L2–L3 vertebrae from each patient were included in the statistical analysis. Receiver operating characteristic (ROC) curve analysis—including calculation of the area under the curve (AUC)—was performed to evaluate the performance of the AI model in diagnosing osteoporosis with respect to the QCT-measured bone density values, and the accuracy, sensitivity, and specificity were calculated. The fit of the bone density values between the AI predictions and the QCT measurements was analyzed with bivariable linear regression. All analyses were two-tailed, and a *p* value of less than 0.001 was considered to indicate statistical significance.

## 3 Results

### 3.1 Statistical comparison of the QCT measurements of the vertebral BMD values

Among the 625 patients, 353 were diagnosed with osteoporosis, 202 with osteopenia, and 70 with normal bone density based on QCT measurements. Of the 305 male patients, 160 had osteoporosis and 108 had osteopenia. Of the 320 female patients, 193 had osteoporosis and 94 had osteopenia. The prevalence of osteoporosis was higher in females than in males, but the difference was not statistically significant (59.1% vs. 53.1%, *p* = 0.312). The BMD values of the T12, L3, and L4 vertebrae measured by QCT showed statistically significant differences between the male and female groups (*p* < 0.001), while the BMD values of the other vertebrae and those measured by different devices did not show significant differences between the two groups (see Table 1).

**Table 1:** Statistical comparison of the QCT measurements of the vertebral BMD values.

Variable	Female *N* = 320	Male *N* = 305	*p* value
Age [mean (SD)]	70.52 (7.98)	71.38 (8.94)	0.204
QCT_L12/L23 (mean (SD))	77.64 (34.94)	80.30 (33.84)	0.335
QCT_T12 (mean (SD))	97.91 (29.72)	77.39 (33.39)	<0.001
QCT_L1 (mean (SD))	84.69 (30.00)	78.91 (38.05)	0.086
QCT_L2 (mean (SD))	82.30 (30.75)	74.46 (39.94)	0.006
QCT_L3 (mean (SD))	83.27 (35.93)	63.36 (31.03)	<0.001
QCT_L4 (mean (SD))	91.75 (30.94)	71.50 (34.06)	<0.001
Neu128_QCT_T12 (mean (SD))	78.18 (34.23)	69.37 (33.71)	0.114
GE_QCT_T12 (mean (SD))	81.54 (34.46)	80.68 (36.05)	0.813
uCT_QCT_L2-L3_AVG (mean (SD))	94.81 (30.01)	77.73 (28.13)	0.022
model 4_QCT_T12 (mean (SD))	73.89 (37.43)	82.21 (32.20)	0.013
BenQ_QCT_L2-L3_AVG (mean (SD))	82.79 (30.52)	78.21 (35.23)	0.351
	QCT detection results (*n*%)	AI detection results (*n*%)	
Normal	35 (10.9)	36 (11.8)	0.312
Osteopenia	96 (30.0)	107 (35.1)	0.242
Osteoporosis	189 (59.1)	162 (53.1)	0.311

*p* value: Indicates statistical significance. Values <0.05 are typically considered significant.

Mean ± SD: Mean values with standard deviation.

### 3.2 Bone density AI diagnostic accuracy for osteoporosis

**Figure 2:**
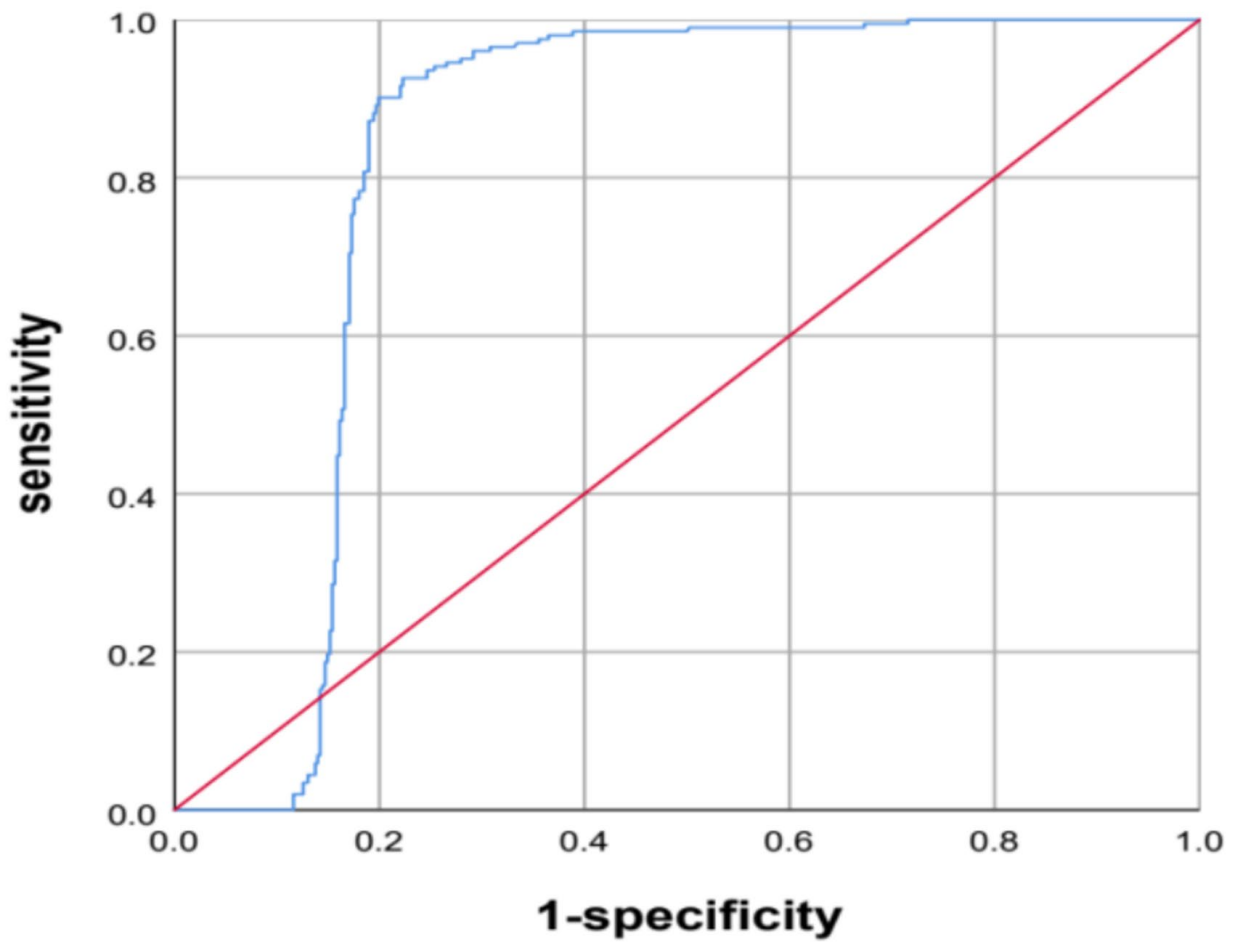
AI diagnosis of osteoporosis ROC curve.

The AI system predicted diagnoses of osteoporosis for 358 patients, reduced bone mass for 197, and normal bone density for 70 patients. The AI predictions and QCT findings did not match for 45 patients. Among these, for 25 patients, the prediction from the AI was worse than the diagnosis based on QCT, including 10 patients for whom the QCT-based diagnosis was normal bone density and the AI predicted reduced bone mass and 15 patients for whom the QCT-based prediction was reduced bone mass and the AI predicted osteoporosis. In 20 patients, the AI results were better than the diagnosis based on QCT, including 10 patients for whom the QCT-based diagnosis was reduced bone mass, while the AI prediction was normal bone density and 10 patients for whom the QCT-based diagnosis was osteoporosis, while the AI prediction was reduced bone mass (see Table 2). The AUC of the AI system in diagnosing osteoporosis was 0.822 (95% CI: 0.787–0.867, *p* < 0.001), with an accuracy of 0.9456, the accuracy is 0.9456, the sensitivity is 0.9601, and the specificity is 0.9270 (see Figure 2).

**Table 2:** Bone density AI diagnostic accuracy for osteoporosis.

Confusion matrix		QCT true value			
		Osteoporosis	Osteopenia	Normal	Sum
AI predicted value	Osteoporosis	343	15	0	358
	Osteopenia	10	177	10	197
	Normal	0	10	60	70
	Sum	353	202	70	625

### 3.3 Comparative analysis of the quantitative QCT-derived and AI-predicted vertebral bone mineral density

A comparative analysis was conducted between the QCT and AI measurements for the quantitative BMD at 5 vertebrae, T12, L1, L2, L3, and L4, as well as the average BMD for L1–L2 or L2–L3, and the consistency between the two methods was assessed. The number of vertebrae, average BMD, and consistency analysis results are presented (Table 3).

**Table 3:** Comparison of the QCT and AI bone density values for different vertebrae.

Classification	T12 (229 cases)	L1 (428 cases)	L2 (614 cases)	L3 (503 cases)	L4 (186 cases)	L12/L23 (625 cases)
QCT	86.71 ± 3.32	81.57 ± 34.66	78.55 ± 35.64	73.81 ± 35.10	79.56 ± 34.24	78.50 ± 32.98
AI	89.24 ± 1.81	83.38 ± 34.03	78.47 ± 33.39	73.09 ± 32.29	73.21 ± 31.14	78.94 ± 34.41
*R* ^2^	0.93	0.95	0.90	0.86	0.95	0.93
*p*	<0.001	<0.001	<0.001	<0.001	<0.001	<0.001cc

**Figure 3:**
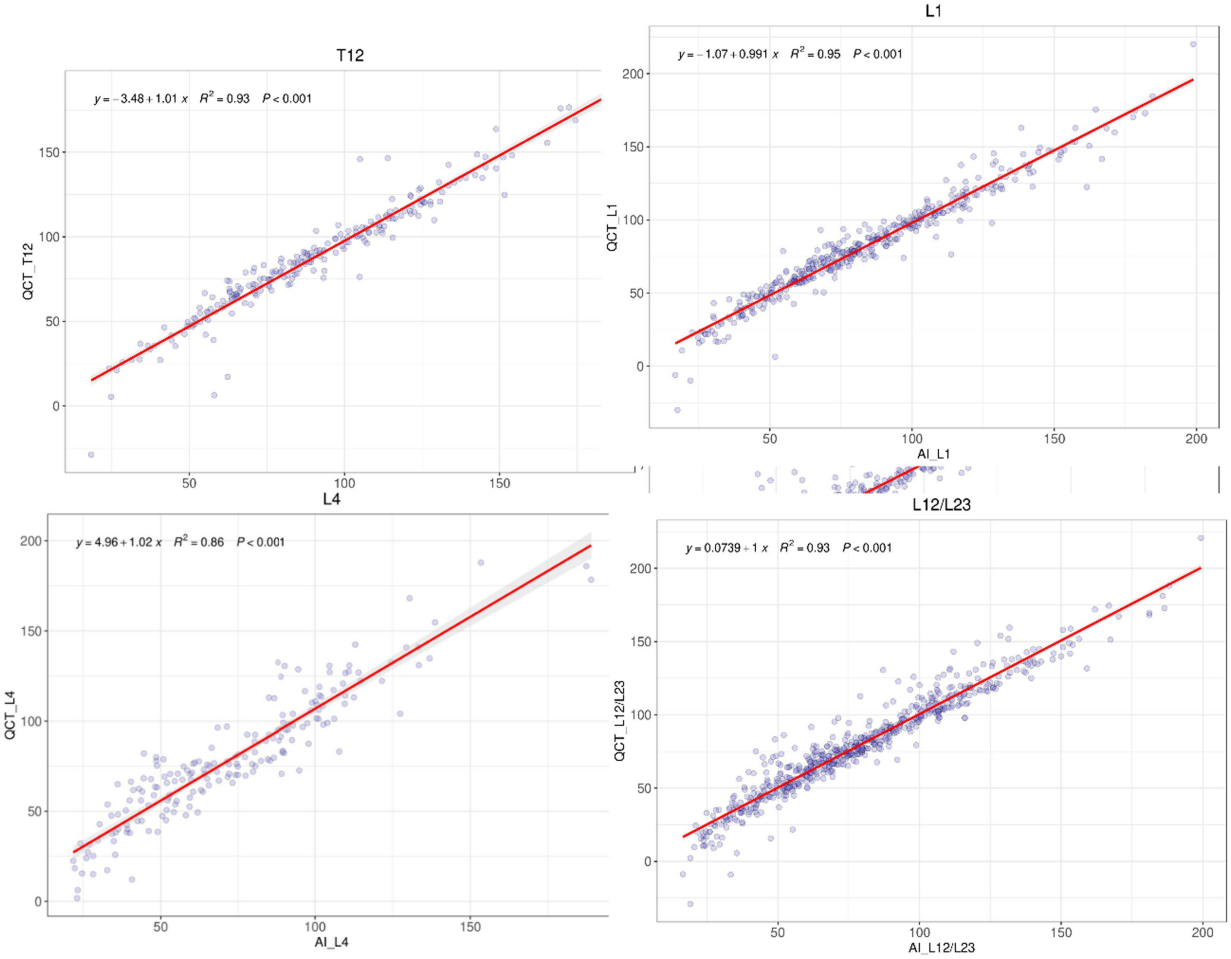
Linear regression analysis of BMD for T12 to L4 Vertebrae between QCT and AI.

The R-squared values for the linear regression fit between the QCT and AI results for the BMD of the T12 to L4 vertebrae were 0.93, 0.95, 0.90, 0.86, and 0.95, respectively. The R-squared value for the average BMD between the L1 and L2 vertebrae or between L2 and L3 vertebrae as measured with QCT and AI was 0.93. The differences in the above comparisons were not significant (see Figure 3).

### 3.4 Comparison of the quantitative QCT and AI lumbar BMD values from different devices

**Figure 4:**
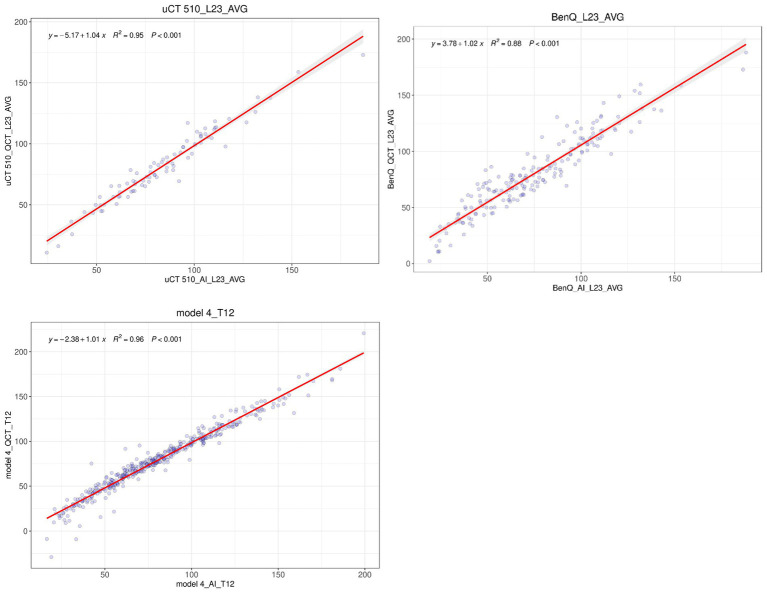
Linear regression analysis of the QCT-derived and AI-predicted values of the mean L1/L2 or mean L2/L3 BMD obtained with three different CT devices.

The consistency in the average BMD measured with QCT and AI at the L1/2 or L2/3 vertebrae on three different CT devices was assessed, and the results are presented in Table 4. The R-squared values for the linear regression fit between the QCT-derived and AI-predicted vertebral BMD values with the GE vCT, Neusoft NeuViz 128, and United Imaging uCT 510 devices were 0.92, 0.95, and 0.95, respectively. The R-squared values for the fit of the AI-predicted vertebral BMD with the third-generation QCT-based values obtained at Mingji Hospital and the fourth-generation QCT-based values obtained at our institution were 0.88 and 0.96, respectively. There were no statistically significant differences in the above comparisons (see Figure 4).

**Table 4:** The quantitative QCT and AI lumbar BMD values from different devices.

Classification	GE (379 case)	Neuviz128 (163 case)	uCT 510 (83 case)	BenQ-QCT3 (192 case)	QCT4 (433 case)
QCT	81.07 ± 35.30	72.51 ± 34.05	81.85 ± 29.35	80.09 ± 33.37	78.43 ± 34.88
AI	79.29 ± 34.41	73.90 ± 31.72	83.95 ± 27.56	74.93 ± 30.82	80.08 ± 33.82
*R* ^2^	0.92	0.95	0.95	0.88	0.96
*v*	<0.001	<0.001	<0.001	<0.001	<0.001

## 4 Discussion

In this study, we utilized QCT measurements of bone mineral density (BMD) as the gold standard to evaluate the performance of a deep learning-based automatic BMD calculation tool. The tool demonstrated robust diagnostic capabilities, achieving an AUC of 0.822 (95% CI: 0.787–0.867, *p* < 0.001) in the qualitative diagnosis of osteoporosis in elderly individuals over 60 years of age, The accuracy, sensitivity, and specificity of the AI model were 94.56, 96.01, and 92.70%, respectively. The deep learning tool also achieved a statistically significant AUC value of 0.822. These metrics underscore the AI model’s proficiency in distinguishing between osteoporotic and non-osteoporotic cases with high precision and sensitivity. Among the 45 patients whose AI-predicted results did not align with QCT measurements, 22 were scanned using a third-generation QCT device, while 23 were scanned with a fourth-generation device. In the third-generation group, the AI tool underestimated BMD in 19 patients, with nine instances of reduced bone mass being misclassified as osteoporosis and 10 instances of normal bone mass being misclassified as reduced bone mass. The discrepancies exceeded 20% in these cases. This significant deviation can be attributed to the absence of silicone padding between the waist and the phantom during QCT scans for nine patients, leading to air gaps between the lumbar spine and the phantom. These gaps introduced artifacts that inflated BMD values post-calibration. In contrast, among the 23 patients scanned with the fourth-generation QCT device, six patients had QCT BMD values below 85 mg/cm^3^, with differences between AI and QCT values remaining under 10%.

The consistency between AI-predicted and QCT-measured BMD values for each vertebra in the T12-L4 region was high, with *R*^2^ values ranging from 0.86 to 0.95. Across different CT models, the consistency between AI and QCT results was also strong, with *R*^2^ values between 0.92 and 0.95, and no statistically significant differences were observed. Notably, the AI software exhibited better consistency with non-synchronized phantom QCT results compared to synchronized phantom QCT results (0.96 vs. 0.88). This discrepancy likely stems from inaccuracies in early synchronized phantom QCT scans due to the lack of silicone padding, which compromised the reliability of the results. To preserve the authenticity of the data, these instances were not excluded from the analysis. However, non-synchronized phantom scans were unaffected by this issue.

The BMD values obtained via AI software differ from those derived through QCT. QCT measures BMD by correcting bone CT values using synchronized or non-synchronized phantoms, focusing solely on the correlation with bone density CT values and disregarding structural changes in trabecular bone. In elderly individuals with significant marrow fat infiltration, this approach can lead to a substantial underestimation of BMD, resulting in QCT-derived BMD values that are approximately 23–27% lower than actual values (Yin et al., 2023; Bredella et al., 2015), In severe cases, this can even yield negative BMD values, as observed in three instances in our study. Utilizing marrow fat quantification calibration or dual-energy CT can produce more accurate and reproducible BMD values than single-energy QCT (Koch et al., 2021). Despite phantom-based CT value corrections, studies using the ESP-145 quantitative phantom have revealed differences in QCT-measured bone density values across CT models from various manufacturers, with more pronounced discrepancies at low-density values (50 mg/cm^3^) (Zhao et al., 2022). Manual adjustment of the position and size of the region of interest (ROI) in QCT measurements, with a maximum height of 9 mm, introduces interobserver variability. In contrast, the AI software segments the vertebrae and employs a nine-grid method to obtain cancellous bone density from different areas, followed by texture analysis to comprehensively determine the BMD values of the corresponding cancellous bone. This automated process effectively mitigates manual measurement errors and enhances repeatability. The BMD values derived from the AI model not only account for changes in bone density but also integrate texture analysis results, reducing reliance on bone density and preventing the generation of negative BMD values. Moreover, the use of QCT phantoms is crucial for AI-based BMD predictions, as regular calibration ensures consistent and comparable measurement results across different devices and over time. The application of these tools and technologies can significantly improve the quality and efficiency of osteoporosis diagnosis and assessment.

## 5 Advantages and limitations of bone density AI

The implementation of automatic bone density AI software tools provides a foundation for opportunistic osteoporosis screening. This approach involves assessing bone density using CT data obtained during routine diagnostic imaging examinations (e.g., chest, abdominal, or lumbar spine CT) as a means of screening for osteoporosis. The advantages of this method are manifold:

- **No additional radiation**: Opportunistic screening does not require additional scans or radiation exposure, allowing bone density assessments to be conducted without increasing the burden on patients.
- **Increased detection rates**: For patients who have not undergone DXA-based screening, leveraging CT data can enhance the diagnosis rate for osteoporosis.
- **Cost-effectiveness**: This method fully utilizes existing imaging data without incurring additional examination costs.
- **Early diagnosis**: Early detection of osteoporosis enables patients to take timely preventive and treatment measures, thereby reducing the risk of fractures.
- **Convenience**: Patients who have already undergone routine CT examinations do not need additional appointments or visits to medical institutions for separate osteoporosis screenings.
- **Rich data**: CT scans provide detailed, three-dimensional bone structure information that can be used for more accurate bone density measurements.

The most commonly used method based on automatic tools, which also happens to be simple and clinically feasible, is to measure the CT values of the anterior 1/3 of the vertebral body. However, this approach measures only a portion of the cancellous bone of the vertebra, which may introduce certain biases. Similar to QCT, the CT values of cancellous bone are influenced by the amount of marrow fat, leading to substantial underestimation of BMD in elderly individuals. Placing a single ROI at the anterior 1/3 of the L1 vertebral body in a sagittal reconstruction image is a fast and effective opportunistic screening technique. The results indicate that BMD is not only related to the T-score of DXA but also to the current and future risk of osteoporotic fractures. While measuring a single vertebra reduces the accuracy and sensitivity in diagnosing osteoporosis, assessing multiple vertebrae strikes a balance between sensitivity and accuracy. Furthermore, combining these measurements with the presence or absence of compression fractures can enhance sensitivity.

AI technology can help mitigate potential CT value differences between different CT scanners, thereby improving diagnostic consistency and accuracy. Acknowledging the potential variations in CT values among scanners is crucial for ensuring diagnostic consistency and accuracy. When comparing diagnostic images across devices or institutions, understanding these differences is vital for clinicians. For instance, Varney et al. used color-coded markers for the CT values of the L1 and L2 vertebrae, employing 145 HU as the threshold to determine abnormal BMD. This method achieved a sensitivity of 92%, specificity of 89%, positive predictive value of 92%, and negative predictive value of 89%, with an accuracy greater than that of manual measurement of the density of the anterior 2/3 of the vertebra (91% vs. 88%) (Morden et al., 2014; Sollmann et al., 2022). Another option is to use the CT value (HU) directly without BMD calibration, although this requires a stable scanner. The CT value of bone depends on the energy distribution of the X-ray spectrum and cannot be standardized through routine water calibration of the CT scanner; thus, it is inherently related to the scanner’s properties. A recent study confirmed this (Park et al., 2024), examining 67,392 CT scans from four different CT scanners, CT scanners from different manufacturers may have varying calibration standards, and even devices from the same manufacturer can produce different CT values based on the model or years of use. AI algorithms can adjust images from different CT scanners to maintain a consistent appearance and intensity distribution, thereby reducing inter-device differences. They can also be employed to develop calibration tools that correct systematic differences between scanners during the image processing stage through machine learning.

## 6 Conclusion

In summary, bone density AI technology holds significant potential and value in the diagnosis and management of osteoporosis and is poised to play a more substantial role in future clinical practice.

## Data Availability

The raw data supporting the conclusions of this article will be made available by the authors without undue reservation.

## References

[ref1] Bredella M. A. Daley S. M. Kalra M. K. Brown J. K. Miller K. K. Torriani M. (2015). Marrow adipose tissue quantification of the lumbar spine by using dual-energy CT and single-voxel 1H MR spectroscopy: a feasibility study. Radiology 277, 230–235. doi: 10.1148/radiol.2015142876, PMID: 25988401 PMC4613879

[ref2] Chen L. Pan Y. Zhong F. Yuan T. J. Wang H. Chen T.. (2022). Using QCT to evaluate bone mineral and abdominal adipose changes in patients with primary hyperparathyroidism and comparing it to DXA for bone status assessment: a retrospective case-control study. Ann. Transl. Med. 10:606. doi: 10.21037/atm-22-1827, PMID: 35722433 PMC9201153

[ref3] Cheng X. Zhao K. Zha X. Du X. Li Y. Chen S.. (2021). Opportunistic screening using low-dose CT and the prevalence of osteoporosis in China: a nationwide, multicenter study. J. Bone Miner. Res. 36, 427–435. doi: 10.1002/jbmr.4187, PMID: 33145809 PMC7988599

[ref4] Clynes M. A. Westbury L. D. Dennison E. M. Kanis J. A. Javaid M. K. Harvey N. C.. (2020). Bone densitometry worldwide: a global survey by the ISCD and IOF. Osteoporos. Int. 31, 1779–1786. doi: 10.1007/s00198-020-05435-8, PMID: 32377806 PMC7115939

[ref5] Hsieh C.-I. Zheng K. Lin C. Mei L. Lu L. Li W.. (2021). Automated bone mineral density prediction and fracture risk assessment using plain radiographs via deep learning. Nat. Commun. 12:5472. doi: 10.1038/s41467-021-25779-x, PMID: 34531406 PMC8446034

[ref6] Imamudeen N. Basheer A. Iqbal A. M. Manjila N. Haroon N. N. Manjila S. (2022). Management of osteoporosis and spinal fractures: contemporary guidelines and evolving paradigms. Clin. Med. Res. 20, 95–106. doi: 10.3121/cmr.2021.1612, PMID: 35478096 PMC9242734

[ref7] Kaesmacher J. Liebl H. Baum T. Kirschke J. S. (2017). Bone mineral density estimations from routine multidetector computed tomography: a comparative study of contrast and calibration effects. J. Comput. Assist. Tomogr. 41, 217–223. doi: 10.1097/RCT.0000000000000518, PMID: 27798444 PMC5359785

[ref8] Kanis J. A. Norton N. Harvey N. C. Jacobson T. Johansson H. Lorentzon M.. (2021). SCOPE 2021: a new scorecard for osteoporosis in Europe. Arch. Osteoporos. 16:82. doi: 10.1007/s11657-020-00871-9, PMID: 34080059 PMC8172408

[ref9] Koch V. Hokamp N. G. Albrecht M. H. Gruenewald L. D. Yel I. Borggrefe J.. (2021). Accuracy and precision of volumetric bone mineral density assessment using dual-source dual-energy versus quantitative CT: a phantom study. Eur. Radiol. Exp. 5:43. doi: 10.1186/s41747-021-00241-1 34608576 PMC8490583

[ref10] McCloskey E. Rathi J. Heijmans S. Blagden M. Cortet B. Czerwinski E.. (2021). The osteoporosis treatment gap in patients at risk of fracture in European primary care: a multi-country cross-sectional observational study. Osteoporos. Int. 32, 251–259. doi: 10.1007/s00198-020-05557-z, PMID: 32829471 PMC7838133

[ref11] Morden N. E. Schpero W. L. Zaha R. Sequist T. D. Colla C. H. (2014). Overuse of short-interval bone densitometry: assessing rates of low-value care. Osteoporos. Int. 25, 2307–2311. doi: 10.1007/s00198-014-2725-2, PMID: 24809808 PMC4210629

[ref12] Park H. Kang W. Y. Woo O. H. Lee J. Yang Z. Oh S. (2024). Automated deep learning-based bone mineral density assessment for opportunistic osteoporosis screening using various CT protocols with multi-vendor scanners. Sci. Rep. 14:25014. doi: 10.1038/s41598-024-73709-w, PMID: 39443535 PMC11499650

[ref13] Shevroja E. Reginster J.-Y. Lamy O. al-Daghri N. Chandran M. Demoux-Baiada A. L.. (2023). Update on the clinical use of trabecular bone score (TBS) in the management of osteoporosis: results of an expert group meeting organized by the European Society for Clinical and Economic Aspects of osteoporosis, osteoarthritis and musculoskeletal diseases (ESCEO), and the international osteoporosis foundation (IOF) under the auspices of WHO collaborating Center for Epidemiology of musculoskeletal health and aging. Osteoporosis Int. 34, 1501–1529. doi: 10.1007/s00198-023-06817-4, PMID: 37393412 PMC10427549

[ref14] Sollmann N. Löffler M. T. El Husseini M. Sekuboyina A. Dieckmeyer M. Rühling S.. (2022). Automated opportunistic osteoporosis screening in routine computed tomography of the spine: comparison with dedicated quantitative CT. J. Bone Miner. Res. Off. J. Am. Soc. Bone Miner. Res. 37, 1287–1296. doi: 10.1002/jbmr.4575, PMID: 35598311

[ref15] Yin H. Lin W. Xie F. He C. Chen T. Zheng G.. (2023). MRI-based vertebral bone quality score for osteoporosis screening based on different osteoporotic diagnostic criteria using DXA and QCT. Calcif. Tissue Int. 113, 383–392. doi: 10.1007/s00223-023-01115-x, PMID: 37493798

[ref16] Zhao Y. Li K. Duanmu Y. Wang L. Xu X. Zhang Y.. (2022). Accuracy, linearity and precision of spine QCT vBMD phantom measurements for different brands of CT scanner: a multicentre study. J. Clin. Densitom. 25, 34–42. doi: 10.1016/j.jocd.2021.02.004, PMID: 33745832

